# Impact of acute temperature and air pollution exposures on adult lung function: A panel study of asthmatics

**DOI:** 10.1371/journal.pone.0270412

**Published:** 2022-06-28

**Authors:** Richard Evoy, Laurel Kincl, Diana Rohlman, Lisa M. Bramer, Holly M. Dixon, Perry Hystad, Harold Bae, Michael Barton, Aaron Phillips, Rachel L. Miller, Katrina M. Waters, Julie B. Herbstman, Kim A. Anderson

**Affiliations:** 1 College of Public Health and Human Sciences, Oregon State University, Corvallis, Oregon, United States of America; 2 Superfund Research Program, Oregon State University, Corvallis, Oregon, United States of America; 3 Biological Sciences Division, Pacific Northwest National Laboratory, Richland, WA, United States of America; 4 Environmental and Molecular Toxicology, Oregon State University, Corvallis, Oregon, United States of America; 5 Computing & Analytics Division, Pacific Northwest National Laboratory, Richland, Washington, United States of America; 6 Icahn School of Medicine at Mount Sinai, New York City, New York, United States of America; 7 Environmental Health Sciences, Mailman School of Public Health, Columbia University, New York City, New York, United States of America; The Ohio State University, UNITED STATES

## Abstract

**Background:**

Individuals with respiratory conditions, such as asthma, are particularly susceptible to adverse health effects associated with higher levels of ambient air pollution and temperature. This study evaluates whether hourly levels of fine particulate matter (PM_2.5_) and dry bulb globe temperature (DBGT) are associated with the lung function of adult participants with asthma.

**Methods and findings:**

Global positioning system (GPS) location, respiratory function (measured as forced expiratory volume at 1 second (FEV_1_)), and self-reports of asthma medication usage and symptoms were collected as part of the Exposure, Location, and Lung Function (ELF) study. Hourly ambient PM_2.5_ and DBGT exposures were estimated by integrating air quality and temperature public records with time-activity patterns using GPS coordinates for each participant (n = 35). The relationships between acute PM_2.5_, DBGT, rescue bronchodilator use, and lung function collected in one week periods and over two seasons (summer/winter) were analyzed by multivariate regression, using different exposure time frames.

In separate models, increasing levels in PM_2.5_, but not DBGT, were associated with rescue bronchodilator use. Conversely DBGT, but not PM_2.5_, had a significant association with FEV_1_. When DBGT and PM_2.5_ exposures were placed in the same model, the strongest association between cumulative PM_2.5_ exposures and the use of rescue bronchodilator was identified at the 0–24 hours (OR = 1.030; 95% CI = 1.012–1.049; p-value = 0.001) and 0–48 hours (OR = 1.030; 95% CI = 1.013–1.057; p-value = 0.001) prior to lung function measure. Conversely, DBGT exposure at 0 hours (β = 3.257; SE = 0.879; p-value>0.001) and 0–6 hours (β = 2.885; SE = 0.903; p-value = 0.001) hours before a reading were associated with FEV_1_. No significant interactions between DBGT and PM_2.5_ were observed for rescue bronchodilator use or FEV_1_.

**Conclusions:**

Short-term increases in PM_2.5_ were associated with increased rescue bronchodilator use, while DBGT was associated with higher lung function (i.e. FEV_1_). Further studies are needed to continue to elucidate the mechanisms of acute exposure to PM_2.5_ and DBGT on lung function in asthmatics.

## Introduction

Ambient air pollution has adverse health effects in susceptible populations, such as those with respiratory diseases [1,2]. Extremes in ambient temperatures may also induce symptoms or exacerbations in asthmatics [3–6]. As climate change continues to cause temperature extremes and create conditions that increase air pollution, it is important to examine the combined effect of increased temperature and air pollution on human health, especially in susceptible populations [2].

Exposure to air pollution, both naturally occurring (wildfires) and manmade (exhaust), is related to increased hospitalization rates, emergency room visits, asthma exacerbations, and reduced lung function in the United States (US) [7–10]. In 2013, asthma was a significant economic burden for Americans and totaled approximately $81.9 billion when both direct and indirect impacts were taken into account [11]. While air pollution has decreased in the US due to efforts to limit greenhouse emissions, regional climate change, and industrial emissions can increase air pollution. One region is the US Pacific Northwest, where an increase in daily maximum temperatures and wildfires are expected to result in higher air pollution levels during the summers [12].

Previous studies have looked at the impacts of air pollution and temperature on asthma exacerbation, bronchodilator use, and lung function [5,6,13–26] independently, but there is a need to explore their interactive relationship relative to respiratory health in individuals with asthma. For studies that examine air pollution and lung function, only the temperature at the exact time of a lung function reading is usually recorded. Only one study we have identified examines temperature, air pollution, and lung function but was focused on healthy adults [22]. Asthmatics are considered to be susceptible to higher levels of air pollution and studies have shown that increased exposure to ambient and personal PM_2.5_ levels can decrease lung function and increase rescue bronchodilator use [15,17–19,22,23,25–30]. Temperature is positively correlated with lung function when personal air pollutant exposure is limited or controlled in asthmatics[3–5,31]. However, studies have also shown the extreme cold or heat can also cause decreases in lung function. [5,6,21,32]. Forced expiratory volume at 1 second (FEV_1_) is a common lung function measurement used to assess obstructive lung physiology and rescue bronchodilator use can act as a surrogate for subjective asthma symptoms. The relationships between air pollution, temperature, and lung function can be further elucidated using spatiotemporal methods coupled with participant global positioning system (GPS) tracking and stationary monitoring of environmental conditions.

During the summer of 2017, the Willamette Valley in Oregon experienced some of the worst air pollution in the US due to smoke from wildfires in Oregon and California [33]. During this time, the Exposure, Location, and Lung Function (ELF) tool was being deployed to participants in Eugene, OR which is located in the Willamette Valley. The ELF tool is an environmental health assessment tool that collects personal data including chemical exposures (passive wristband sampler), location (mobile phone GPS) and lung function measures (spirometry) as well as self-reported information (through the ELF Tracker application at the time of measures) [34]. The ELF tool relies on community-engaged research which represents a novel way to quickly gather data with participants collecting their own data. While some loss of potential accuracy may be present, researchers do gain a broader representation of participation. In addition, as recent public health emergencies, like the COVID-19 pandemic has shown, there are times when research can be conducted in collaboration with communities when researchers do not have access to the communities or medical facilities. In this panel analysis, we examine how exposure metrics from public data for PM_2.5_ and temperature are associated with the lung function (i.e. FEV_1_) of participants and rescue bronchodilator use.

## Materials and methods

### Study design

This panel study used data from 35 physician-diagnosed asthmatics who enrolled in the ELF study from 2017 to 2018. Participants were recruited from an allergy clinic in Eugene, OR. Participants were interviewed about symptoms and asthma history before enrollment to ensure that participants met eligibility requirements. Eligible participants were given the Asthma Control Test [35]. All participants gave informed consent. The ethics board of Oregon State University approved the study (protocol #8058).

Participants received an Android cell phone pre-loaded with the ELF Tracker application, passive chemical wristband sampling devices [36], and a mobile spirometer [37]. Participants were enrolled for a total of 14 days; 7 consecutive days during the summer and 7 consecutive days during the winter. Of the 35 participants, 6 participated for 7 days while the other 29 completed 14 days. Participants were enrolled in three different time periods consisting of 89 days in total: *Summer 2017* (September 29-November 3); *Winter 2018* (February 6-April 10); and *Summer 2018* (September 7-October 12).

Twice a day (morning and evening) participants self-recorded three FEV_1_ measurements using the Spirotel spirometer (Medical International Research, eHealth minilab, v1) and answered a short questionnaire about their protocol compliance, asthma medication use, and symptoms (**S1 File**). The participants carried the ELF phone, which cataloged and transmitted location (GPS) and spirometry data to the research team in real-time.[34] When enrolled, the participant completed a health questionnaire and was given the ELF toolkit to start the study the following morning. For this study, only data from the ELF phone, the questionnaire and spirometry data were utilized. Results from the silicone wristbands are not presented.

### Spirometry calibration, training, and processing

American Thoracic Society (ATS) certified personnel conducted all training and spirometry calibration. All spirometers underwent calibration with a 3L syringe before distribution. All calibration tests were within +/- 0.15 L (5%) [38]. Each participant measured their FEV_1_ in triplicate, on consecutive days, once in the morning and once in the evening. Participants were provided verbal instructions in the use of the spirometer and coached until a valid reading was obtained during enrollment. The ELF toolkit included a User Guide for each participant, along with a one-page abbreviated sheet of instructions for taking valid readings. These readings were transmitted from the spirometer to the ELF phone and stored in a cloud. For both morning and evening measures, a valid FEV_1_ reading was defined as the two highest FEV_1_ measures at one-time point that were also within +/- 0.15 L of one another [39]. Valid readings were used as described below in the analyses and linked to exposure measures by the date and time of the reading.

### Self-reported symptoms and asthma medication usage

Following each spirometry measurement, participants completed a brief survey documenting asthma medication and rescue bronchodilator use, and asthma symptoms (wheezing, coughing, shortness of breath, chest tightness or pain) within the last 6 hours. All participants were instructed to continue daily asthma medication and use their emergency bronchodilator medication as typically self-administered throughout the study.

### Location data

Valid, time-stamped questionnaire and lung function data were used to identify the start and end dates/times for each participant within their study period. Missing GPS data were estimated by reviewing location coordinates immediately before and after the missing data. If these were the same latitude and longitude, the missing location data were estimated to be the same. If the data before and after the missing data contained different latitudes and longitudes, the timestamp, latitude, and longitude on either side of the gap were used to extrapolate the missing data using linear interpolation. The estimated data were visually checked for spatiotemporal errors.

### Exposure metrics

#### Air quality

Hourly PM_2.5_ data (parameter code 88502) from land-based air pollution monitors were downloaded from the Environmental Protection Agency (EPA) air quality index data [40] for Oregon for the years 2017 and 2018 (downloaded 03/18/2020). The GPS and EPA hourly data were rounded to the nearest hour and linked by date, time, and nearest location. The distance between each GPS point and air pollution monitor location was calculated. GPS data that were more than 50 kilometers away from a monitor were assigned as missing for PM_2.5_ exposure. This was done to remove potential outliers while ensuring the homes of the participants were assigned exposures. The number of hours spent at each GPS location was multiplied by the PM_2.5_ measurements to generate hourly time-weighted average exposure values. For analysis, the PM_2.5_ exposure at the time of the lung function reading is indicated as 0 hour. The hourly time-weighted exposures were averaged to estimate exposure windows for 0–6, 0–12, 0–24, and 0–48 hours prior to the FEV_1_ reading. Although the EPA provides ozone and nitrogen dioxide (NO2) data, not all monitoring locations measure these. In Oregon, most of the monitors that measure these air pollutants were located near Portland, OR which is over 150 km from Eugene, OR where the participants live.

#### Temperature

National Oceanic and Atmospheric Administration (NOAA) hourly local climatological data [41] for dry bulb globe temperature (DBGT) were downloaded for Oregon for 2017 and 2018 (downloaded 03/20/2020). The same aforementioned interpolation methodology applied for the location was used to generate missing hourly DBGT hourly measurements. The GPS data and DBGT hourly data were rounded to the nearest hour and linked by date, time, and nearest location. GPS data more than 50 kilometers away from a monitor were assigned as missing for DBGT exposure. The exposures were averaged to create hourly average DBGT exposure. For analysis, the exposure at the time of the lung function reading is indicated as 0 hour. The hourly DBGT was averaged to estimate exposure windows for 0–6, 0–12, 0–24, and 0–48 hours prior to the FEV_1_ recording.

### Statistical analysis

To evaluate how rescue bronchodilator use and measures of FEV_1_ were associated with acute exposures to PM_2.5_ and DBGT, each exposure (0 hour, 0–6, 0–12, 0–24, 0–48 hours prior to reading) was independently run in unadjusted and adjusted mixed-effect models. The participant was included in the model as a random effect to account for the potential dependence of their measurements PM_2.5_ and DBGT exposures were also placed in the same adjusted model and checked for significant interactions. R (Version 3.5.0) was used for all data management and statistical analyses using linear and logistic mixed-effects models to account for within-subject variability in the outcome variables. P-values less than 0.05 were considered statistically significant.

Adjusted mixed-effects models were run with *a priori* chosen list of covariates based on findings from other panel studies that examined PM_2.5_ or DBGT exposures and lung function [16,17,19–21,23,24,42–45]. Covariates included were age (linear and quadratic), morning or evening reading (morning/evening), normal daily asthma medication usage (yes/no), and time period (*Summer 2017/Winter 2018/Summer 2018*).

#### Rescue bronchodilator use

To evaluate if PM_2.5_ was associated with rescue bronchodilator use the effects of PM_2.5_ and DBGT were evaluated in separate mixed-effects logistic regression models with the participant included as a random effect. Neither splines nor interquartile ranges (IQR) PM_2.5_ exposure transformations were found to significantly improve model fit. The 0 hour and cumulative PM_2.5_ exposures were each placed in models with DBGT exposures to determine whether exposure to PM_2.5_ changed while controlling for DBGT. Cross product terms were placed in the models to determine if any significant interaction with the air quality and temperature occurred that impacted bronchodilator use. Multicollinearity between DBGT and PM_2.5_ exposure metrics was checked using the variance inflation factor.

#### Lung function (FEV_1_)

Readings when rescue bronchodilator medication was self-administered within 6 hours of the reading were excluded from the analyses for FEV_1_ to minimize residual pharmacological effects on the FEV_1_ measures. To evaluate the impact of acute exposures on FEV_1_, the same variables and approach used in the logistic models (described above) were assessed in mixed-effects linear regression models. Similar to previous panel studies of lung function, FEV_1_ was not transformed [16,17,19–21,23,24,26,28]. Adjusted models were then run with covariates selected *a priori*. Models were not significantly improved with splines nor IQR exposures for PM_2.5_ and DBGT. Cross product terms were then placed in the models to determine if any significant interaction occurred. Multicollinearity between DBGT and PM_2.5_ exposure metrics was checked using the variance inflation factor.

#### Sensitivity analyses

Multiple sensitivity analyses were conducted to evaluate various assumptions used in the mixed-effects regression models. The cut-off distance between EPA air monitors and GPS coordinates was changed to 25 kilometers instead of 50. Hourly time-weighted averages were generated for all PM_2.5_ exposures using this new cut-off distance. The results from all the models, both linear and logistic, were compared to see how the models handled the larger number of missing values and the corresponding coefficients. The participant variable was run as a fixed-effect, instead of a random-effect, in all models to examine individual level changes in FEV_1_. Finally, a stratified analyses by summer and winter time periods were examined to determine how the PM_2.5_ and DBGT effects differ by season.

## Results

### Participant and lung function readings

The average age of the participants was 49 years old (range: 21–74 years; SD = 15.5). There were 28 women and 7 men in the study. The majority of participants were Caucasian (85%). Additional details about the study population can be found in **Table 1**. There were 23 participants enrolled in *Summer 2017*, 32 in *Winter 2018*, and 9 in *Summer 2018*. A total of 968 ELF measurements (spirometry and asthma symptom question responses) were collected (**Table 2**) and 625 (64.6%) were used for the analysis of FEV_1_, after excluding readings that were missing FEV_1_ values, used rescue bronchodilator medication, or were missing questionnaire data. For the rescue bronchodilator use analysis, 846 (87.4%) readings were used, following the exclusion of readings without questionnaire data.

**Table 1:** ELF participant demographic information.

Variable	Count (% or Standard Deviation)
**Sex: N (%)**	
Female	28 (81%)
Male	7 (19%)
**Age [years]: mean (SD), (Range)**	49 (15.5 SD), 21–74
**Height [inches]: mean (SD)**	66 (3.4 SD)
**Race: n (%)**	
White	30 (85%)
Black / African American	1 (3%)
American Indian/Alaska Native	1 (3%)
More than One Race	1 (3%)
Unknown/Other	2 (6%)
**Ethnicity: n (%)**	
Hispanic or Latino	3 (8%)
Not Hispanic or Latino	26 (75%)
Decline to answer	6 (17%)
**Years with asthma: mean (SD), (Range)**	35.4 (19.0), 1–72
**Asthma Control Test: mean (SD), (Range)**	20.9 (3.0), 13–25

**Table 2:** Lung function and exposure metrics by time period.

	Time Period		
	Summer 2017	Winter 2018	Summer 2018
**Number of Participants**	23	32	9
**ELF Measurements**			
Total Measurements Recorded	341	490	137
Missing Questionnaire	27 (7.9%)	65 (13.3%)	20 (14.6%)
Rescue Bronchodilator Used	28 (8.2%)	34 (6.9%)	6 (4.4%)
Missing FEV1 Reading	61 (17.9%)	78 (15.9%)	24 (17.5%)
Valid Measure*	225 (65.9%)	313 (63.9%)	87 (63.5%)
**FEV**_**1**_ **Measures**			
Mean (ml)	2,389	2,468	2,509
Interquartile Range (ml)	1,870–2,820	2,025–2,885	2,150–2,780
**Number of GPS points**	28,727	31,731	9,170
**Average Minutes Between GPS points**	9.45 mins	12.2 mins	12.2 mins
**Average Distance from Air Pollution Monitors (Kilometers)**	12.7 KM	8.94 KM	10.7 KM
**Average Monitor PM**_**2.5**_ **(μg/m**^**3**^**)**	29.0 μg/m^3^	4.33 μg/m^3^	9.56 μg/m^3^
**Average Distance from Temperature Monitors (Kilometers)**	18.5 KM	14.8 KM	17.4 KM
**Average Dry Bulb Globe Temperature (F)**	66.7°F	43.2°F	72.3°F

* No rescue bronchodilator, no missing questionnaire, and valid FEV1 reading. Used in linear regression models.

### Spatiotemporal exposure metrics for PM_2.5_ and DBGT

Of the 66,708 GPS points collected, 323 (0.5%) and 1,198 (1.8%) were over 50 km from the nearest stationary air pollution and DBGT monitor. Details about the interpolation of GPS data can be found in **S1 File**. Of the 968 total readings for FEV_1_ for all participants, only 2 (0.2%) readings were missing PM_2.5_ exposures and only 10 (1.0%) were missing DBGT exposure estimates. Of these, only 2 (0.2%) readings were missing both PM_2.5_ and DBGT exposure estimates. Details of these metrics can be found in **Table 2**.

### Impacts of PM_2.5_ and DBGT on rescue bronchodilator use

There were no significant associations between DBGT and rescue bronchodilator use in adjusted logistic regression models. Cumulative PM_2.5_ exposures of 0–12, 0–24, and 0–48 hours prior to readings were significantly associated with rescue bronchodilator use. The strongest association between cumulative PM_2.5_ and bronchodilator medication use was identified at the 0–24 (OR = 1.030; 95% CI = 1.013–1.048; p-value<0.001) and 0–48 (OR = 1.036; 95% CI = 1.016–1.056; p-value<0.001) hours prior to spirometer use. Results from exposures in unadjusted and adjusted logistic models can be found in **S1 File** for PM_2.5_ and DBGT.

When temperature was taken into account with PM_2.5_, the strongest association between cumulative PM_2.5_ exposures and the use of rescue bronchodilator was identified at the 0–24 (OR = 1.030; 95% CI = 1.012–1.049; p-value = 0.001) and 0–48 hours (OR = 1.030; 95% CI = 1.013–1.057; p-value = 0.001) prior to spirometer use (**Table 3**). With PM_2.5_ and DBGT each in the model, no significant associations between DBGT and rescue bronchodilator use remained. All 25 combinations of DBGT and PM_2.5_ exposure durations were tested for interactions (multiplicative or additive) but none were statistically significant.

**Table 3:** Adjusted odds ratios of rescue bronchodilator use for PM_2.5_ (1-μg/m^3^) and temperature (1°F) in the same model.

	PM_2.5_ (1 μg/m^3^)			Dry Bulb Globe Temperature (1°F)		
Time	OR	95% CI	p-value	OR	95% CI	p-value
0	1.016	0.988–1.046	0.261	1.017	0.975–1.061	0.438
0–6	1.012	0.999–1.026	0.071	1.017	0.967–1.055	0.652
0–12	1.021	1.005–1.036	**0.007**	0.987	0.941–1.035	0.582
0–24	1.030	1.012–1.049	**0.001**	1.009	0.945–1.077	0.794
0–48	1.035	1.013–1.057	**0.001**	1.018	0.945–1.097	0.635

Logistic model

Rescue_Meds~PM_2.5_+DBGT+Medication+MornOrEven+TimePeriod+age+age^2+(1|Participant_ID).

Rescue_Meds = 1 (used rescue bronchodilator within 6 hours of reading) or 0 (didn’t use within 6 hours).

MornOrEven = Morning (04:00–12:59) or Evening readings (13:00–1:00).

Medication = 1 (used non-rescue asthma medication) or 0 (didn’t use).

TimePeriod = Summer 2017, Winter 2018, or Summer 2018.

### Impacts of PM_2.5_ and DBGT on FEV_1_

In the adjusted models for PM_2.5_ and FEV_1_ there was only one significant association for 0–12 hours (b = 0.835; SE = 0.411; p-value = 0.042). In the adjusted models for DBGT and FEV_1_, all exposure windows (Hour 0, 0–6, 0–24, 0–48) except 0–12 were found to have significant associations with FEV_1_. DBGT hour 0 (p-value<0.001) and 0–6 (p-value<0.001) had the biggest impact on FEV_1_.The results from exposures in unadjusted and adjusted linear regression models can be found in **S1 File** for PM_2.5_ and DBGT.

When PM_2.5_ and DBGT were each in the model, both hour 0 and hours 0–6 for DBGT remained highly significant (p-value< = 0.001), while all PM_2.5_ exposures were not significant (**Table 4**). All adjusted models reported increases in FEV_1_ as PM_2.5_ levels increased regardless of whether DBGT was taken into account or not (**S1 File**). No cross-product interactions between DBGT and PM_2.5_ were significant. All the cross-product terms were showed that as PM2.5 and DBGT increased, FEV1 increased.

**Table 4:** Adjusted change in respiratory function per increase in PM_2.5_ (1-μg/m^3^) and temperature (1°F) in the same model.

	PM_2.5_ (1 μg/m^3^)			Dry Bulb Globe Temperature (1°F)		
Time	β	SE (β)	p-value	β	SE (β)	p-value
0	0.745	0.627	0.234	3.527	0.879	**<0.001**
0–6	0.750	0.366	0.076	2.885	0.903	**0.001**
0–12	0.757	0.418	0.070	1.212	1.005	0.228
0–24	0.449	0.490	0.359	2.418	1.272	0.057
0–48	0.223	0.586	0.704	2.991	1.446	**0.038**

Adjusted linear model

fev1_ml~PM_2.5_+DBGT+Medication+MornOrEven+TimePeriod+age+age^2+(1|Participant_ID).

MornOrEven = Morning (04:00–12:59) or Evening readings (13:00–1:00).

Medication = 1 (used non-rescue asthma medication) or 0 (didn’t use).

TimePeriod = Summer 2017, Winter 2018, or Summer 2018.

### Sensitivity and stratified analyses

Results from the sensitivity and stratified analyses can be found in the **S1 File**. When GPS points over 25 km away from an air pollution monitor were removed from calculations for hourly time-weighted average exposure to PM_2.5_, the results remained relatively similar to the original findings. DBGT was only significant for Hour 0 and 0–6 hours before a reading while PM_2.5_ was only significant for 0–6 and 0–12 hours before a reading. Regardless of the season, when PM_2.5_ or DBGT increased, FEV_1_ increased. For rescue bronchodilator use, PM_2.5_ was the only exposure to be significantly associated with increased use. Using the participant as a fixed-effect did not significantly change the findings from the linear and logistic regression.

Stratified analyses revealed no significant associations between PM_2.5_, DBGT, and FEV_1_ during the winter (**S1 File**). DBGT was significantly associated with FEV_1_ during the summer (**S1 File**). For rescue bronchodilator use, PM_2.5_ exposure was significantly associated with increased use during the summer but not during the winter (**Table 5**). For the winter, only PM_2.5_ 0–6 hours before a reading was found to have a significant association with rescue bronchodilator use. All of the PM2.5 exposures during the winter were found to decrease the likelihood that participants used rescue bronchodilator medication as PM2.5 levels increased (**Table 6**).

**Table 5:** Adjusted odds ratios of rescue bronchodilator usage for PM_2.5_ and DBGT in the same model for participants in the summer.

	PM_2.5_ (1 μg/m^3^)			Dry Bulb Globe Temperature (1°F)		
Time	OR	95% CI	p-value	OR	95% CI	p-value
0	1.032	0.997–1.068	0.076	1.013	0.946–1.084	0.712
0–6	1.018	1.001–1.035	**0.033**	0.981	0.912–1.056	0.615
0–12	1.027	1.008–1.046	**0.006**	0.946	0.874–1.024	0.173
0–24	1.033	1.010–1.056	**0.001**	0.990	0.883–1.110	0.860
0–48	1.041	1.014–1.069	**0.003**	0.991	0.872–1.125	0.885

Logistic model

Rescue_Meds~PM_2.5_+DBGT+Medication+MornOrEven +age+age^2+(1|Participant_ID).

Rescue_Meds = 1 (used rescue bronchodilator within 6 hours of reading) or 0 (didn’t use within 6 hours) MornOrEven = Morning (04:00–12:59) or Evening readings (13:00–1:00).

Medication = 1 (used non-rescue asthma medication) or 0 (didn’t use).

**Table 6:** Adjusted odds ratios of rescue bronchodilator usage for PM_2.5_ and DBGT in the same model for participants in the winter.

	PM_2.5_ (1 μg/m^3^)			Dry Bulb Globe Temperature (1°F)		
Time	OR	95% CI	p-value	OR	95% CI	p-value
0	0.724	0.478–1.096	0.127	0.994	0.928–1.066	0.870
0–6	0.502	0.288–0.875	**0.015**	0.970	0.901–1.045	0.422
0–12	0.734	0.490–1.100	0.134	0.961	0.889–1.038	0.308
0–24	0.854	0.581–1.256	0.423	0.997	0.912–1.090	0.943
0–48	0.881	0.563–1.379	0.579	1.013	0.912–1.124	0.816

Logistic model

Rescue_Meds~PM_2.5_+DBGT+Medication+MornOrEven +age+age^2+(1|Participant_ID).

Rescue_Meds = 1 (used rescue bronchodilator within 6 hours of reading) or 0 (didn’t use within 6 hours).

MornOrEven = Morning (04:00–12:59) or Evening readings (13:00–1:00).

Medication = 1 (used non-rescue asthma medication) or 0 (didn’t use).

## Discussion

### Health effects of PM_2.5_ and DBGT exposure

This study found that as PM_2.5_ concentrations increased so did FEV_1_ measurements, independent of the model used. This is similar to findings from other air pollution panel studies that did not analyze lung function readings when rescue bronchodilator medication was used, or that analyzed those readings separately [27,30,46]. This association may partially be explained by the exclusion of FEV_1_ readings with rescue bronchodilator use. We did find that the average exposure to PM_2.5_ was higher when participants reported the use of their rescue bronchodilators. Since we excluded FEV_1_ readings measured within 6 hours of rescue bronchodilator use to minimize confounding by residual drug-induced bronchodilation, this removed some of the higher PM_2.5_ readings from the linear regression analysis looking at the association of PM_2.5_ and FEV_1_.

### Comparisons to other panel studies

Previous panel studies of repeated lung function measures and exposure to ambient PM_2.5_ have reported conflicting results, with several reporting no association [16,17,19,20], and others reporting negative associations [18,22–24,30]. Wu et al. reported that estimated PM_2.5_ effects were generally stronger in the presence of high temperatures than in low temperatures.[22] Although Wu et al. suggested that ambient air pollution and temperature may interact synergistically to adversely lower FEV_1_, our study did not find any interactions. As we mentioned above, the removal of lung function readings taken within 6 hours of rescue bronchodilator use may have obscured the impact of PM_2.5_ on FEV_1_ and any possible interactions.

This study illustrated that the use of a rescue bronchodilator is associated with acute previous cumulative exposure to ambient PM_2.5_ but not elevated temperatures. Therefore, evaluating impacts of PM_2.5_ using FEV_1_ in asthmatics is problematic, as to self-manage their health they may require rescue bronchodilator use obscuring any effect that PM_2.5_ may have on FEV_1_ measures. Studies in healthy adults, who may be less susceptible, may further elucidate impacts on FEV_1_ from acute exposures to PM_2.5_, although such an approach may be less sensitive as healthy adults may have a more stable FEV1. The PM_2.5_ exposure latencies from this study showed that the PM_2.5_ concentrations at the time a spirometer reading was taken was never significantly associated with rescue bronchodilator use. Our study adds to the growing body of literature on the impact of latent air pollution exposures on asthmatics. Our stratified analyses by summer and winter showed that the impact of PM_2.5_ was different depending on the season. During the summer, increased PM_2.5_ cumulative exposures were all significantly associated with an increased risk of rescue bronchodilator use. All of the ORs for PM_2.5_ during the winter were below 1 which showed that participants were less likely to use rescue bronchodilators as PM_2.5_ levels increased.

### Application of findings

Our findings suggest that cumulative, ambient air pollution levels impact rescue bronchodilator use in asthmatics most during summer time. As climate change continues to worsen air quality (duration and concentration) in this region, as has been seen in the summer of 2020 [47], public health and medical management of the health impacts for vulnerable populations is critical.

### Potential limitations of the study

We acknowledge several limitations of the study. The participants were a convenience sample recruited at an asthma clinic. The applicability of the results in this study could be limited due to several factors. We acknowledge that the small sample size (n = 35) could have limited our ability to detect significant changes in FEV_1_. Additionally, our study was comprised mostly of females (81%) and white (85%) which limits the generalizability of the findings to the overall adult asthmatic population. The exposure metrics used in the study were dependent on participant compliance (i.e. carrying the phone with them), but every individual who participated twice in our study recorded 84 lung function tests which we feel is an acceptable number of tests to evaluate changes over time and their validity. Overall compliance was high, as the average percentage of interpolated data by hour was less than 8% (**S1 Table in S1 File**).

The exposure metrics generated for this study represent ambient PM_2.5_ and temperature levels that may not reflect the actual exposures for individuals with time spent inside. We decided to use publicly available sources of exposure data from the EPA that were used in our previous study [34] but the distribution of GPS location data varied considerably from this study. Our sensitivity analyses also showed that reducing the cut-off point between monitors and participant GPS locations by half did not significantly change the results. The decision to adjust all models by age, time of reading, time period, and participant identification number were made *a priori*. Participants continued to use asthma medication throughout the study and rescue bronchodilator medication was self-reported for approximately 5% of FEV_1_ readings taken.

There are several strengths of the study. They include: (1) repeated observations for participants across different seasons and different times of day; (2) wide distribution of air quality levels due to wildfire smoke in the summer of 2017; (3) study with asthmatics and; (4) community engaged research that highlights illustrates how participants can correctly and accurately collect their own data.

## Conclusion

As climate change continues to increase temperatures and consequently air pollution levels, it is important to study how environmental conditions impact susceptible populations. This study investigates how environmental factors can impact human health using community gathered data. Our research identifies a caveat in measuring lung function in asthmatics since the exposure causes respiratory distress. More research is needed to conclusively determine the short-term health effects of air pollution and/or temperature to better understand the cumulative impact these exposures can have on the health of vulnerable communities.

## Supporting information

S1 Data (XLSX)

S1 File (DOCX)

## Abbreviations

PM_2.5_: Fine Particulate Matter
DBGT: Dry Bulb Globe Temperature
ELF: Exposure Location and Lung Function
FEV_1_: Forced Expiratory Volume at 1 second
GPS: Global Position System
EPA: Environmental Protection Agency
NOAA: National Oceanic and Atmospheric Administration
