# Supplementary material for: Impact of acute temperature and air pollution exposures on adult lung function: A panel study of asthmatics
Source: PLoS One. 2022 Jun 28;17(6):e0270412. doi: 10.1371/journal.pone.0270412 (PMC9239441; doi:10.1371/journal.pone.0270412)
Supplement: S1 File — (DOCX) [file pone.0270412.s002.docx]

**Supplemental Information**

**ELF app questionnaire**

**Questions after every spirometry reading**

1. *Did you use your asthma medication with the last 6 hours before or after this test?*

1 = Yes

0 = No

If yes, when?

Time (Hh:mm)

1. *Did you use your rescue inhaler within the last 6 hours before or during this test?*

1= Yes

0 = No

If yes, when?

Time (Hh:mm)

1. *Were you around anyone who was smoking before or during this test?*

1 = Yes

0 = No

**Questions only asked for morning spirometry readings**

1. *Did you wear the wristband for an entire day (since yesterday morning)?*

1 = Yes

0 = No

NA = not applicable

[If No] Please select the reason you did not wear the wristband

1 = Forgot

2 = Inconvenient

3 = Left in my general location (home, office, etc.)

4 = Device malfunction (call study coordinator)

5 = Other (please record in daily diary)

1. *Please enter the wristband ID number (on the bag) for the wristband you are putting on*

[Assigned wristband IDs are shown and participant selects the one shown on the bag of the wristband.]

**Questions only asked for evening spirometry readings**

1. *Did you experience any of the following asthma symptoms today? Mark all that apply.*

Wheezing

1 = Yes

0 = No

Coughing

1 = Yes

0 = No

Shortness of breath

1 = Yes

0 = No

Chest tightness or pain

1 = Yes

0 = No

1. *How many times did you use your asthma medication today?*

0 = 0 times

1 = 1 time

2 = 2 times

3 = 3 or more times

1. *How many times did you use your rescue inhaler today?*

0 = 0 times

1 = 1 time

2 = 2 times

3 = 3 or more times

1. *Did you carry the phone on your person today?*

1 = Yes

0 = No

[If No] Please select the reason why you didn’t carry the phone

1 = Forgot

2 = Inconvenient

3 = Left in my general location (home, office, etc.)

4 = Device malfunction (call study coordinator)

5 = Other (please record in daily diary)

1. *How much time did you spend outdoors today?*

0 = no time

1 = less than 1 hour

2 = from 1 up to 8 hours

3 = from 8 up to 12 hours

4 = from 12 up to 24 hours

1. *How much time did you spend in transit today?*

0 = no time

1 = less than 1 hour

2 = from 1 up to 8 hours

3 = from 8 up to 12 hours

4 = from 12 up to 24 hours

**If participant missed a morning reading**

1. *Whoops! Missed morning read. What happened?*

1 = Forgot

2 = left spirometer elsewhere and missed the reading

3 = Asthma symptoms prevented test

4 = Device malfunction

5 = Other (please record in daily diary)

**If participant missed an afternoon reading**

1. *Whoops! Missed evening reading. What happened?*

1 = Forgot

2 = left spirometer elsewhere and missed the reading

3 = Asthma symptoms prevented test

4 = Device malfunction

5 = Other (please record in daily diary)

**GPS data**

Approximately 95,711 GPS coordinates were originally recorded for ELF participants for all three time periods. The start and end dates for participant enrollment in each study period were used to limit GPS data to only those within the start and end dates. Participants were given the ELF toolkit one day prior to enrollment so GPS coordinate data that were a day before the start date for all participants was used. This resulted in 29,003 (~33.3%%) GPS coordinates being removed since they were outside the study time period, leaving 66,708 (~69.7%). The resulting GPS coordinates were then aggregated by hour in order to calculate the total number of hours covered and identify missing GPS data. The start and end dates for participants was then used to calculate the total number of observations hours if GPS coordinates were always being recorded. Approximately 12,825 hours of GPS data were expected to be recorded if there were no missing GPS data. We have 9,905 (~77.2%) hours of participant GPS location data. Using linear regression, the missing hourly data was interpolated to generate the 2,920 (~22.8%) hours of missing GPS data. **Supplemental Table 1** gives a break down by hour of the real vs interpolated data.

**Supplemental Table 1: Real and Interpolated data by Hour for all participants and time periods**

| Hour | Number of Real GPS data | Number of Interpolated Hourly GPS data | Percent of Data Interpolated |
| --- | --- | --- | --- |
| 0:00 (Midnight) | 1,411 | 109 | 7.7% |
| 1:00 | 1,056 | 150 | 14.2% |
| 2:00 | 891 | 172 | 19.3% |
| 3:00 | 856 | 214 | 25.0% |
| 4:00 | 862 | 221 | 25.6% |
| 5:00 | 982 | 216 | 22.0% |
| 6:00 | 1,852 | 188 | 10.2% |
| 7:00 | 5,266 | 137 | 2.6% |
| 8:00 | 5,935 | 79 | 1.3% |
| 9:00 | 3,613 | 68 | 1.9% |
| 10:00 | 3,013 | 63 | 2.1% |
| 11:00 | 3,187 | 78 | 2.4% |
| 12:00 | 4,078 | 77 | 1.9% |
| 13:00 | 3,133 | 103 | 3.3% |
| 14:00 | 2,604 | 113 | 4.3% |
| 15:00 | 2,695 | 124 | 4.6% |
| 16:00 | 4,350 | 98 | 2.3% |
| 17:00 | 4,631 | 95 | 2.1% |
| 18:00 | 4,793 | 86 | 1.8% |
| 19:00 | 3,142 | 99 | 3.2% |
| 20:00 | 2,702 | 91 | 3.4% |
| 21:00 | 2,428 | 93 | 3.8% |
| 22:00 | 1,872 | 84 | 4.5% |
| 23:00 | 1,356 | 103 | 7.6% |

Of the 2,920 hours of interpolated GPS data, 2053 (~70.3%) GPS points had the same latitude and longitude as real GPS data indicating no movement. Approximately, 867 (~29.7%) of the interpolated GPS data had different latitude and longitude indicating the participants were moving. The new expanded GPS coordinate data (69,618 observations) was used for creating and calculating exposures metrics.

**Exposure metrics**

**Air pollution**

Haversine formula was used to calculate distance between individual GPS coordinates and all EPA air pollution monitors in Oregon based on Time (H:M) and Date (M/D/Y). The nearest air monitor for a GPS point was then used to represent exposure. Air monitors that were more than 50 kilometers from a GPS point were considered missing. Only 323 (0.4%) GPS points were more than 50 km from an air pollution monitor. For the sensitivity analysis, we limited the GPS points to only those that were less than 25 kilometers from an air monitor. Approximately 6,256 (9.4%) GPS points were more than 25 kilometers from an air monitor.

The time spent at each GPS point was calculated using the GPS timestamps from chronically sorted data by participant and time period. Using the time spent, and the exposures for each GPS point, we calculated hourly time-weighted average exposures for the entire time participants were enrolled by time period (Summer vs Winter). This process was used to calculate 0-6, 0-12, 0-24 and 0-48 hours of exposure for each hour a participant. One or a combination of these cumulative exposure durations were used in previous panel studies for air pollution and respiratory health.^1–12,13,14^ Simple moving averages of exposures, that did not take into account time spent, were generated as well to use to see how the different exposure methods compared.

**Temperature**

The NOAA temperature data for all of Oregon was limited to readings that had the same start and end dates as the participant GPS data. This resulted in 148,272 hourly temperature readings for all monitors in Oregon but approximately 8,798 (5.9%) were missing measurements. Using linear regression, the temperature for these missing temperature measurements was interpolated.

Haversine formula was used to calculate distance between individual GPS coordinates and all NOAA temperature monitors in Oregon. The nearest air monitor for a GPS point was then used to represent exposure. Of the 66,617 GPS points, 1,198 (~1.8%) were more than 50 kilometers away and were considered missing. For the sensitivity analysis, 5,842 (~8.8%) GPS points were between 25 and 50 kilometers away and were considered missing. Simple moving averages of exposures were generated but not time-weighted averages. These were then used to calculate 0-6, 0-12, 0-24 and 0-48 hours of exposure for each participant. One or a combination of these cumulative exposure durations were used in previous panel studies for temperature and respiratory health [7,15–17].

**Air pollution and temperature panel studies**

Thus far, we have only been able to identify one study that examines cumulative exposures prior to a lung function reading for both temperature and air pollution [7]. In this study, they used Hour 0, 0-3, 0-6, 0-12, 0-18, 0-24 and 0-7 day averages for both. We decided to use Hour 0, 0-6, 0-12, 0-24 and 0-48 because it would allow us to compare our results with other panel studies [1,5,10–12,18,19] that looked at various cumulative exposure readings for temperature and/or air pollution.

**Analyses**

**Rescue bronchodilator use analysis**

**Supplemental Table 2** and **Supplemental Table 3** show the unadjusted and adjusted logistic model outputs for the impact of PM_2.5_ on rescue bronchodilator medication usage. The adjusted model results are reported in the primary manuscript.

**Supplemental Table 2:** **Unadjusted and adjusted odds ratios of rescue bronchodilator medication usage for PM_2.5_ (1-µg/m^3^)**

|  | PM2.5  (1 µg/m^3^) | | | Adjusted* PM2.5  (1 µg/m^3^) | | |
| --- | --- | --- | --- | --- | --- | --- |
| Time | OR | 95% CI | p-value | OR | 95% CI | p-value |
| 0 | 1.011 | 0.986-1.036 | 0.393 | 1.018 | 0.990-1.047 | 0.217 |
| 0-6 | 1.008 | 0.996-1.019 | 0.182 | 1.012 | 0.999-1.026 | 0.070 |
| 0-12 | 1.015 | 1.013-1.018 | **<0.001** | 1.019 | 1.004-1.034 | **0.005** |
| 0-24 | 1.025 | 1.023-1.028 | **<0.001** | 1.030 | 1.013-1.048 | **<0.001** |
| 0-48 | 1.029 | 1.012-1.047 | **<0.001** | 1.036 | 1.016-1.056 | **<0.001** |

Logistic model:

Rescue_Meds~Time+Medication+MornOrEven+TimePeriod+age+age^2+(1|Participant_ID)

Rescue_Meds = 1 (used rescue bronchodilator within 6 hours of reading) or 0 (didn’t use within 6 hours)
MornOrEven = Morning (04:00-12:59) or Evening readings (13:00-1:00)
Medication = 1 (used non-rescue asthma medication) or 0 (didn’t use)
TimePeriod = Summer 2017, Winter 2018, or Summer 2018

**Supplemental Table 3: Unadjusted and adjusted odds ratios of rescue bronchodilator medication usage for DBGT (1°F)**

|  | Dry Bulb Globe Temperature  (1°F) | | | Adjusted* Dry Bulb Globe Temperature (1°F) | | |
| --- | --- | --- | --- | --- | --- | --- |
| Time | OR | 95% CI | p-value | OR | 95% CI | p-value |
| 0 | 1.001 | 0.979-1.023 | 0.935 | 1.020 | 0.978-1.064 | 0.355 |
| 0-6 | 1.005 | 0.986-1.025 | 0.578 | 1.013 | 0.969-1.058 | 0.571 |
| 0-12 | 1.001 | 0.982-1.021 | 0.903 | 0.999 | 0.955-1.046 | 0.993 |
| 0-24 | 0.998 | 0.976-1.020 | 0.842 | 1.046 | 0.985-1.111 | 0.143 |
| 0-48 | 0.997 | 0.976-1.020 | 0.818 | 1.062 | 0.992-1.138 | 0.086 |

Logistic model:

Rescue_Meds~Time+Medication+MornOrEven+TimePeriod+age+age^2+(1|Participant_ID)

Rescue_Meds = 1 (used rescue bronchodilator within 6 hours of reading) or 0 (didn’t use within 6 hours)
MornOrEven = Morning (04:00-12:59) or Evening readings (13:00-1:00)
Medication = 1 (used non-rescue asthma medication) or 0 (didn’t use)
TimePeriod = Summer 2017, Winter 2018, or Summer 2018

**Lung function (FEV_1_) analysis**

**Supplemental Table 4** and **Supplemental Table 5** show the unadjusted and adjusted linear model outputs for the impact of PM2.5 on FEV_1_ lung function measures. The adjusted model results are reported in the primary manuscript.

**Supplemental Table 4: Unadjusted and adjusted variations in respiratory function per increase of PM_2.5_ (1-µg/m^3^)**

|  | PM2.5  (1 µg/m^3^) | | | Adjusted* PM2.5  (1 µg/m^3^) | | |
| --- | --- | --- | --- | --- | --- | --- |
| Time | β | SE (β) | p-value | β | SE (β) | p-value |
| 0 | 1.043 | 0.685 | 0.128 | 1.245 | 0.703 | 0.077 |
| 0-6 | 0.718 | 0.390 | 0.066 | 0.841 | 0.407 | **0.040** |
| 0-12 | 0.763 | 0.421 | 0.070 | 0.923 | 0.441 | **0.036** |
| 0-24 | 0.546 | 0.481 | 0.256 | 0.755 | 0.507 | 0.136 |
| 0-48 | 0.371 | 0.572 | 0.517 | 0.603 | 0.608 | 0.321 |

Unadjusted linear model: fev1_ml~PM_2.5_+Participant_ID
*Adjusted linear model: fev1_ml~Time+Medication+MornOrEven+TimePeriod+Age+Age^2+(1|Participant_ID)
MornOrEven = Morning (04:00-12:59) or Evening readings (13:00-1:00)
Medication = 1 (used non-rescue asthma medication) or 0 (didn’t use)
TimePeriod = Summer 2017, Winter 2018, or Summer 2018

**Supplementary Table 5: Unadjusted and adjusted variations in respiratory function per increase in DBGT (1°F)**

|  | Dry Bulb Globe Temperature  (1°F) | | | Adjusted* Dry Bulb Globe Temperature (1°F) | | |
| --- | --- | --- | --- | --- | --- | --- |
| Time | β | SE (β) | p-value | β | SE (β) | p-value |
| 0 | 0.444 | 0.469 | 0.345 | 3.662 | 0.872 | **<0.001** |
| 0-6 | 0.038 | 0.419 | 0.927 | 2.988 | 0.903 | **<0.001** |
| 0-12 | -0.336 | 0.434 | 0.439 | 1.514 | 0.993 | 0.128 |
| 0-24 | -0.165 | 0.479 | 0.730 | 2.743 | 1.222 | **0.025** |
| 0-48 | -0.234 | 0.479 | 0.626 | 3.141 | 1.390 | **0.024** |

Unadjusted linear model: fev1_ml~DBGT+Participant_ID

*Adjusted linear model: fev1_ml~Time+Medication+MornOrEven+TimePeriod+Age+Age^2+(1|Participant_ID)
MornOrEven = Morning (04:00-12:59) or Evening readings (13:00-1:00)
Medication = 1 (used non-rescue asthma medication) or 0 (didn’t use)
TimePeriod = Summer 2017, Winter 2018, or Summer 2018

**Sensitivity analyses**

Sensitivity Analyses were run using exposure values from GPS points that were less than 25 kilometers away from an air pollution monitor instead of 50 kilometers. The cut-off point of 50 km used in the analysis presented in manuscript was done in order to capture exposures at all participants’ homes. Approximately 80% of the GPS points were within 25 kilometers of the temperature and air quality monitors. **Supplemental Table 6** and **Supplemental Table 7** show the results from the sensitivity analyses for both analysis models when both exposures were in the same model. **Supplemental Table 8 and Supplemental Table 9** show the results from using Participant as a fixed-effect instead of a random-effect in logistic and linear regression models.

**Supplemental Table 6: Sensitivity Analysis for adjusted odds ratios of rescue bronchodilator medication usage for PM_2.5_ (1-µg/m^3^) and DBGT (1°F) in the same model**

|  | PM_2.5_  (1 µg/m^3^) | | | Dry Bulb Globe Temperature  (1°F) | | |
| --- | --- | --- | --- | --- | --- | --- |
| Time | OR | 95% CI | p-value | OR | 95% CI | p-value |
| 0 | 1.028 | 0.997-1.060 | 0.081 | 1.016 | 0.973-1.060 | 0.465 |
| 0-6 | 1.015 | 1.000-1.030 | **0.047** | 1.010 | 0.966-1.056 | 0.663 |
| 0-12 | 1.022 | 1.006-1.038 | **0.007** | 0.988 | 0.942-1.035 | 0.604 |
| 0-24 | 1.029 | 1.010-1.049 | **0.003** | 1.013 | 0.949-1.081 | 0.698 |
| 0-48 | 1.036 | 1.013-1.060 | **0.002** | 1.012 | 0.947-1.098 | 0.609 |

Logistic model:

Rescue_Meds~PM_2.5_+DBGT+Medication+MornOrEven+TimePeriod+age+age^2+(1|Participant_ID)

Rescue_Meds = 1 (used rescue bronchodilator within 6 hours of reading) or 0 (didn’t use within 6 hours)
MornOrEven = Morning (04:00-12:59) or Evening readings (13:00-1:00)
Medication = 1 (used non-rescue asthma medication) or 0 (didn’t use)
TimePeriod = Summer 2017, Winter 2018, or Summer 2018

**Supplemental Table 7: Sensitivity analysis for adjusted variations in FEV_1_ per increase in PM_2.5_ (1-µg/m^3^) and DBGT (1°F) in the same model**

|  | PM_2.5_  (1 µg/m^3^) | | | Dry Bulb Globe Temperature  (1°F) | | |
| --- | --- | --- | --- | --- | --- | --- |
| Time | β | SE (β) | p-value | β | SE (β) | p-value |
| 0 | 0.953 | 0.700 | 0.173 | 3.537 | 0.876 | **<0.001** |
| 0-6 | 0.772 | 0.405 | 0.060 | 2.890 | 0.902 | **0.001** |
| 0-12 | 0.844 | 0.447 | 0.060 | 1.224 | 1.003 | 0.222 |
| 0-24 | 0.484 | 0.528 | 0.358 | 2.421 | 1.271 | 0.057 |
| 0-48 | 0.253 | 0.631 | 0.688 | 2.982 | 1.446 | **0.039** |

Adjusted linear model: fev1_ml~PM2.5+DBGT+Medication+MornOrEven+TimePeriod+age+age^2+(1|Participant_ID)

MornOrEven = Morning (04:00-12:59) or Evening readings (13:00-1:00)
Medication = 1 (used non-rescue asthma medication) or 0 (didn’t use)
TimePeriod = Summer 2017, Winter 2018, or Summer 2018

**Impact of using participant as a fixed-effect**

**Supplemental Table 8: Sensitivity analysis for adjusted odds ratios of rescue bronchodilator medication use for PM_2.5_ (1-µg/m^3^) and DBGT (1°F) in the same model using participant as a fixed-effect**

|  | PM_2.5_  (1 µg/m^3^) | | | Dry Bulb Globe Temperature  (1°F) | | |
| --- | --- | --- | --- | --- | --- | --- |
| Time | OR | 95% CI | p-value | OR | 95% CI | p-value |
| 0 | 1.019 | 0.987-1.052 | 0.216 | 1.037 | 0.989-1.082 | 0.138 |
| 0-6 | 1.015 | 1.000-1.031 | 0.056 | 1.032 | 0.985-1.081 | 0.184 |
| 0-12 | 1.024 | 1.008-1.042 | **0.005** | 1.004 | 0.955-1.055 | 0.883 |
| 0-24 | 1.033 | 1.012-1.058 | **0.003** | 1.046 | 0.976-1.123 | 0.205 |
| 0-48 | 1.035 | 1.012-1.063 | **0.004** | 1.061 | 0.980-1.152 | 0.148 |

Logistic model:

Rescue_Meds~PM_2.5_+DBGT+Medication+MornOrEven+TimePeriod+age+age^2+Participant_ID

Rescue_Meds = 1 (used rescue bronchodilator within 6 hours of reading) or 0 (didn’t use within 6 hours)
MornOrEven = Morning (04:00-12:59) or Evening readings (13:00-1:00)
Medication = 1 (used non-rescue asthma medication) or 0 (didn’t use)
TimePeriod = Summer 2017, Winter 2018, or Summer 2018

**Supplemental Table 9: Sensitivity analysis for adjusted variations in FEV1 per increase in PM_2.5_ (1-µg/m^3^) and DBGT (1°F) in the same model using participant as a fixed-effect**

|  | PM_2.5_  (1 µg/m^3^) | | | Dry Bulb Globe Temperature  (1°F) | | |
| --- | --- | --- | --- | --- | --- | --- |
| Time | β | SE (β) | p-value | β | SE (β) | p-value |
| 0 | 0.991 | 0.648 | 0.127 | 3.316 | 0.867 | **<0.001** |
| 0-6 | 0.776 | 0.390 | **0.047** | 2.794 | 0.891 | **0.002** |
| 0-12 | 0.861 | 0.430 | **0.046** | 1.138 | 0.992 | 0.252 |
| 0-24 | 0.561 | 0.501 | 0.263 | 2.235 | 1.258 | 0.076 |
| 0-48 | 0.316 | 0.600 | 0.599 | 2.717 | 1.430 | 0.058 |

Adjusted linear model: fev1_ml~PM_2.5_+DBGT+Medication+MornOrEven+TimePeriod+age+age^2+Participant_ID

MornOrEven = Morning (04:00-12:59) or Evening readings (13:00-1:00)
Medication = 1 (used non-rescue asthma medication) or 0 (didn’t use)
TimePeriod = Summer 2017, Winter 2018, or Summer 2018

**Stratified analyses**

**Lung function (FEV_1_) analysis**

As the time period in which a reading was taken (Summer vs. Winter) was a significant covariate in the adjusted linear models we ran stratified analyses to see how the time period covariates impacted the effects of temperature and PM on lung function (FEV_1_). **Supplemental Table 10** and **Supplemental Table 11** show the results from the stratified analyses for both analysis models when both exposures were in the same model.

**Supplemental Table 10: Adjusted variations in FEV_1_ per increase in PM_2.5_ (1-µg/m^3^) and DBGT (1°F) for participants in the winter**

|  | PM_2.5_  (1 µg/m^3^) | | | Dry Bulb Globe Temperature  (1°F) | | |
| --- | --- | --- | --- | --- | --- | --- |
| Time | β | SE (β) | p-value | β | SE (β) | p-value |
| 0 | -1.652 | 5.733 | 0.773 | 2.251 | 1.317 | 0.087 |
| 0-6 | -2.006 | 4.712 | 0.670 | 1.067 | 1.416 | 0.451 |
| 0-12 | 0.346 | 5.023 | 0.945 | -0.735 | 1.588 | 0.643 |
| 0-24 | 3.527 | 7.757 | 0.627 | 0.108 | 1.852 | 0.955 |
| 0-48 | 5.615 | 8.772 | 0.552 | -0.546 | 2.160 | 0.800 |

Adjusted linear model: fev1_ml~PM2.5+DBGT+Medication+MornOrEven+age+age^2+(1|Participant_ID)

MornOrEven = Morning (04:00-12:59) or Evening readings (13:00-1:00)

Medication = 1 (used non-rescue asthma medication) or 0 (didn’t use)

**Supplemental Table 11: Adjusted variations of FEV_1_ per increase in PM_2.5_ (1-µg/m^3^) and DBGT (1°F) for participants in the summer**

|  | PM_2.5_  (1 µg/m^3^) | | | Dry Bulb Globe Temperature  (1°F) | | |
| --- | --- | --- | --- | --- | --- | --- |
| Time | β | SE (β) | p-value | β | SE (β) | p-value |
| 0 | 0.722 | 0.676 | 0.286 | 4.624 | 1.176 | **<0.001** |
| 0-6 | 0.692 | 3.392 | 0.077 | 4.689 | 1.496 | **<0.001** |
| 0-12 | 0.847 | 0.442 | 0.055 | 1.568 | 1.553 | 0.313 |
| 0-24 | 0.459 | 0.537 | 0.393 | 2.677 | 2.149 | 0.213 |
| 0-48 | 0.153 | 0.649 | 0.814 | 3.552 | 2.520 | 0.159 |

Adjusted linear model: fev1_ml~PM2.5+DBGT+Medication+MornOrEven+age+age^2+(1|Participant_ID)

MornOrEven = Morning (04:00-12:59) or Evening readings (13:00-1:00)
Medication = 1 (used non-rescue asthma medication) or 0 (didn’t use)

**References**

1. Lagorio S, Forastiere F, Pistelli R, Iavarone I, Michelozzi P, Fano V, et al. Air pollution and lung function among susceptible adult subjects: a panel study. Environ Health Glob Access Sci Source. 2006;5: 11–12. doi:10.1186/1476-069X-5-11

2. Silkoff PE, Zhang L, Dutton S, Langmack EL, Vedal S, Murphy J, et al. Winter air pollution and disease parameters in advanced chronic obstructive pulmonary disease panels residing in Denver, Colorado. J Allergy Clin Immunol. 2005;115: 337–344. doi:10.1016/j.jaci.2004.11.035

3. Ni Y, Wu S, Ji W, Chen Y, Zhao B, Shi S, et al. The exposure metric choices have significant impact on the association between short-term exposure to outdoor particulate matter and changes in lung function: Findings from a panel study in chronic obstructive pulmonary disease patients. Sci Total Environ. 2016;542: 264–270. doi:10.1016/j.scitotenv.2015.10.114

4. Peacock JL, Anderson HR, Bremner SA, Marston L, Seemungal TA, Strachan DP, et al. Outdoor air pollution and respiratory health in patients with COPD. Thorax. 2011;66: 591–596. doi:10.1136/thx.2010.155358

5. Hartog JJ de, Ayres JG, Karakatsani A, Analitis A, Brink H ten, Hameri K, et al. Lung function and indicators of exposure to indoor and outdoor particulate matter among asthma and COPD patients. Occup Environ Med. 2010;67: 2–10. doi:10.1136/oem.2008.040857

6. Jansen Karen L., Larson Timothy V., Koenig Jane Q., Mar Therese F., Fields Carrie, Stewart Jim, et al. Associations between Health Effects and Particulate Matter and Black Carbon in Subjects with Respiratory Disease. Environ Health Perspect. 2005;113: 1741–1746. doi:10.1289/ehp.8153

7. Wu S, Deng F, Hao Y, Wang X, Zheng C, Lv H, et al. Fine particulate matter, temperature, and lung function in healthy adults: Findings from the HVNR study. Chemosphere. 2014;108: 168–174. doi:10.1016/j.chemosphere.2014.01.032

8. Dales R, Chen L, Frescura AM, Liu L, Villeneuve PJ. Acute effects of outdoor air pollution on forced expiratory volume in 1 s: a panel study of schoolchildren with asthma. Eur Respir J. 2009;34: 316–323. doi:10.1183/09031936.00138908

9. Jacobson L da SV, Hacon S de S, Castro HA de, Ignotti E, Artaxo P, Ponce de Leon ACM. Association between fine particulate matter and the peak expiratory flow of schoolchildren in the Brazilian subequatorial Amazon: a panel study. Environ Res. 2012;117: 27–35. doi:10.1016/j.envres.2012.05.006

10. Adam M, Schikowski T, Carsin AE, Cai Y, Jacquemin B, Sanchez M, et al. Adult lung function and long-term air pollution exposure. ESCAPE: a multicentre cohort study and meta-analysis. Eur Respir J. 2015;45: 38–50. doi:10.1183/09031936.00130014

11. Baccarelli AA, Zheng Y, Zhang X, Chang D, Liu L, Wolf KR, et al. Air pollution exposure and lung function in highly exposed subjects in Beijing, China: a repeated-measure study. Part Fibre Toxicol. 2014;11. doi:10.1186/s12989-014-0051-7

12. Int Panis L, Provost EB, Cox B, Louwies T, Laeremans M, Standaert A, et al. Short-term air pollution exposure decreases lung function: a repeated measures study in healthy adults. Environ Health. 2017;16. doi:10.1186/s12940-017-0271-z

13. Jung KH, Torrone D, Lovinsky-Desir S, Perzanowski M, Bautista J, Jezioro JR, et al. Short-term exposure to PM2.5 and vanadium and changes in asthma gene DNA methylation and lung function decrements among urban children. Respir Res. 2017;18: 63. doi:10.1186/s12931-017-0550-9

14. Santos UP, Garcia MLSB, Braga ALF, Pereira LAA, Lin CA, de André PA, et al. Association between Traffic Air Pollution and Reduced Forced Vital Capacity: A Study Using Personal Monitors for Outdoor Workers. PLoS ONE. 2016;11: 1–12. doi:10.1371/journal.pone.0163225

15. Li S, Baker PJ, Jalaludin BB, Marks GB, Denison LS, Williams GM. Ambient temperature and lung function in children with asthma in Australia. Eur Respir J. 2014;43: 1059–1066. doi:10.1183/09031936.00079313

16. Collaco JM, Appel LJ, McGready J, Cutting GR. The relationship of lung function with ambient temperature. PLOS ONE. 2018;13: e0191409. doi:10.1371/journal.pone.0191409

17. Lin Z, Gu Y, Liu C, Song Y, Bai C, Chen R, et al. Effects of ambient temperature on lung function in patients with chronic obstructive pulmonary disease: A time-series panel study. Sci Total Environ. 2018;619–620: 360–365. doi:10.1016/j.scitotenv.2017.11.035

18. Chen C-H, Wu C-D, Chiang H-C, Chu D, Lee K-Y, Lin W-Y, et al. The effects of fine and coarse particulate matter on lung function among the elderly. Sci Rep. 2019;9: 14790. doi:10.1038/s41598-019-51307-5

19. Delfino Ralph J., Staimer Norbert, Tjoa Thomas, Gillen Dan, Kleinman Michael T., Sioutas Constantinos, et al. Personal and Ambient Air Pollution Exposures and Lung Function Decrements in Children with Asthma. Environ Health Perspect. 2008;116: 550–558. doi:10.1289/ehp.10911
